# Analysis of Optic Chiasmal Compression Caused by Brain Tumors Using Optical Coherence Tomography Angiography

**DOI:** 10.1038/s41598-020-59158-1

**Published:** 2020-02-07

**Authors:** Ga-In Lee, Kyung-Ah Park, Sei Yeul Oh, Doo-Sik Kong

**Affiliations:** 1 Department of Ophthalmology, Samsung Medical Center, Sungkyunkwan University School of Medicine, Seoul, Korea; 2 Department of Neurosurgery, Endoscopic Skull Base Surgery Clinic, Brain Tumor Center, Samsung Medical Center, Sungkyunkwan University School of Medicine, Seoul, Korea

**Keywords:** Optic nerve diseases, Diagnostic markers

## Abstract

We have quantitatively evaluated the macular and peripapillary microvascular changes in eyes with chiasmal compression caused by brain tumors compared with healthy control eyes using optical coherence tomography angiography (OCT-A) and correlated them with other ocular parameters. This cross-sectional study involved the analysis of 36 eyes of 36 patients with chiasmal compression and age and refractive error-matched 35 healthy control eyes. OCT-A was used to generate microvascular images of the superficial and deep retinal capillary plexus (SRCP, DRCP) and the radial peripapillary capillary (RPC) segment in the macula and peripapillary areas. Automated segmentation and vessel density measurements facilitated the analysis of each layer. Macular OCT-A analysis revealed a significant reduction in vessel density in the SRCP (*P* = 0.004) of the nasal quadrant (*P* < 0.001) and in the same quadrant of the DRCP (*P* = 0.019) in the eyes with chiasmal compression compared with the control eyes. The RPC segment vessel density has also been significantly reduced in the eyes with chiasmal compression (*P* < 0.001). The RPC segment and the SRCP vessel densities were correlated with the peripapillary retinal nerve fiber layer and the ganglion cell layer complex thicknesses. The RPC segment and the nasal quadrant SRCP and the DRCP vessel densities were correlated with visual field defect. Significant microvascular alterations have been detected in the eyes with chiasmal compression compared with the control eyes. This study confirmed that chiasmal compression caused by brain tumors not only induced a loss of ganglion cells but also resulted in intra-retinal microvascular changes.

## Introduction

Visual dysfunction is a common symptom of various tumors involving the sellar area. A growing tumor compresses the retinal ganglion cell (RGC) axons, resulting in visual field defects and reduced visual acuity. Optical coherence tomography (OCT) has been used to evaluate morphological changes in the retina and the peripapillary retinal nerve fiber layer (pRNFL) in compressive optic neuropathy^1–4^. It is well known that pRNFL thinning^1,2,5^ and ganglion cell loss^4,6,7^ detected by OCT are correlated with visual field defects in compressive optic neuropathies^4,6,7^. Recent advances in OCT-Angiography (OCT-A) as a new imaging modality to characterize the three-dimensional vascular structures of the retinal layers and peripapillary areas, have led to the identification of microvascular changes in optic neuropathies such as glaucoma^8^, ischemic optic neuropathies^9,10^, and inflammatory optic neuropathies^11^. The studies reported a significant correlation between microvascular alterations and visual functions, such as visual acuity^10,11^ and visual field^10,12^. To date, only a single case-series has been conducted to investigate the microvascular changes in eyes with chiasmal compression using OCT-A^13^. The objective of the current study has been to analyze the microvascular changes involving macula and optic nerve head using OCT-A and to correlate microvascular changes with clinical parameters in chiasmal compression.

## Results

**Figure 1:**
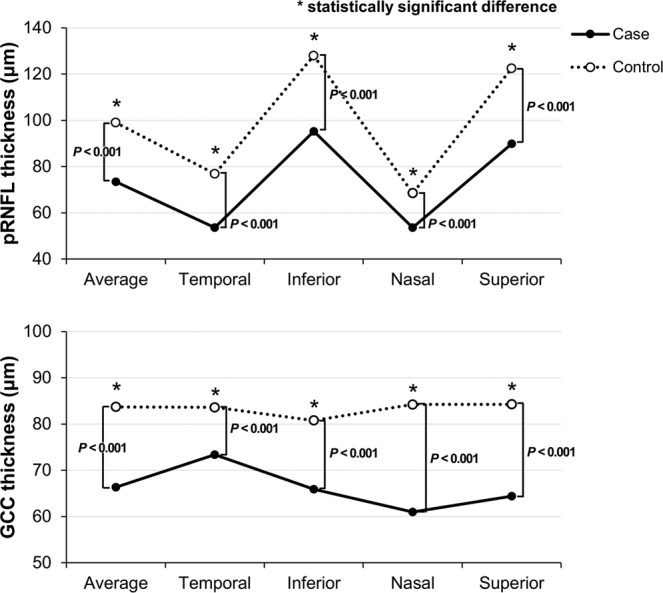
Comparison of the peripapillary retinal nerve fiber layer (pRNFL) and ganglion cell layer complex thickness (GCC) in healthy controls and patients with chiasmal compression. The thickness of the pRNFL and GCC was significantly lower in the case group than in the control group, including the average values and the four-quadrant values. Asterisks indicate statistically significant differences between the two groups.

The study included a total of 36 eyes of 36 patients with chiasmal compression and 35 eyes of healthy controls. These patients exhibited chiasmal compression due to a pituitary adenoma in the sellar and suprasellar areas (27 patients), craniopharyngioma in the suprasellar area (two patients), meningiomas in the tuberculum sella (six patients), and a pituicytoma in the pituitary stalk (one patient). The mean largest diameter of the tumor was 25.82 ± 10.32 mm (range, 6–60 mm) and the duration of the visual symptoms ranged from 1 to 24 months. All patients underwent transsphenoidal tumor resections. No statistical differences were found in age, gender, or spherical equivalent (SE) refractive errors between the two groups (Table 1). The best corrected visual acuity (BCVA) and visual field defects varied significantly between the two groups. The thickness of pRNFL and ganglion cell layer complex (GCC) was significantly lower in the patient group than in the control group (all *P* < 0.001), including the average values and the four-quadrant values **(**Table 2, Fig. 1**)**.

**Table 1:** Demographics of patients with chiasmal compression due to brain tumor and healthy control group.

Variable	Chiasmal compression group, n = 36	Healthy Control group, n = 35	*P* Value
	Mean ± SD	Mean ± SD	
Sex, male/female	22/14	15/20	0.258
Age, y	52 ± 14	50 ± 12	0.6
BCVA, logMAR	0.30 ± 0.63	0.0 ± 0.0	0.007*
Spherical equivalent, diopters	−0.89 ± 1.87	−1.21 ± 1.63	0.44
IOP, mm Hg	15.22 ± 3.23	16.11 ± 2.87	0.310
Visual field^†^, MD (dB), [range]	−14.46 ± 9.85 [−31.29 to 0.35]	0.12 ± 1.15	<0.001*
Symptom duration, month, [range]	3.64 ± 2.93 [1 to 24]	NA	NA

SD = standard deviation; BCVA = best corrected visual acuity; IOP = intraocular pressure; MD = mean deviation; NA = not applicable. *Statistically significant. ^†^Humphrey Field Analyzer using the 30-2 SITA-standard protocol.

**Table 2:** Comparison of intraretinal layer thickness in the healthy control and patients with chiasmal compression.

	Average	Temporal	Inferior	Nasal	Superior
**pRNFL thickness**, **μm**					
Case	73.44 ± 14.71	53.58 ± 13.99	95.22 ± 23.40	53.58 ± 10.76	89.94 ± 22.63
Control	99.09 ± 7.12	76.91 ± 12.47	127.97 ± 13.29	68.49 ± 9.25	122.51 ± 13.73
*P* Value	<0.001*	<0.001*	<0.001*	<0.001*	<0.001*
**GCC thickness**, **μm**					
Case	66.35 ± 10.87	73.35 ± 10.28	65.87 ± 10.38	61.00 ± 14.09	64.39 ± 12.78
Control	83.71 ± 5.39	83.62 ± 5.26	80.79 ± 5.32	84.24 ± 5.02	84.26 ± 6.18
*P* Value	<0.001*	<0.001*	<0.001*	<0.001*	<0.001*

Data are given as mean ± standard deviation. GCC = ganglion cell layer complex; pRNFL = peripapillary retinal nerve fiber layer. *Statistically significant.

### Microvascular densities in the parafoveal area

Comparison of the macular microvascular density between the eyes with chiasmal compression and the control eyes indicated that the average vessel density of the superficial retinal capillary plexus (SRCP) in the eyes with chiasmal compression was significantly lower than in the control eyes after adjusting for age and SE (46.8% ± 2.7% vs. 48.7% ± 2.6%, *P* = 0.004). When compared by sectors, a significant difference existed only in the nasal quadrant (44.6% ± 2.5% in the eyes with chiasmal compression vs. 47.5% ± 2.6% in the control eyes, *P* < 0.001). The difference in the average vessel density of the deep retinal capillary plexus (DRCP) in the macular area was not significantly different between the eyes with chiasmal compression and the control eyes. However, when compared according to sectors, the DRCP vessel density in the nasal quadrant was significantly lower in the eyes with chiasmal compression than in the nasal quadrant of the control eyes after adjusting for age and SE (46.3% ± 3.6% vs. 48.3% ± 2.9%, *P* = 0.019). The differences in DRCP vessel density in other sectors were not statistically significant.

### Microvascular densities in the peripapillary area

**Figure 2:**
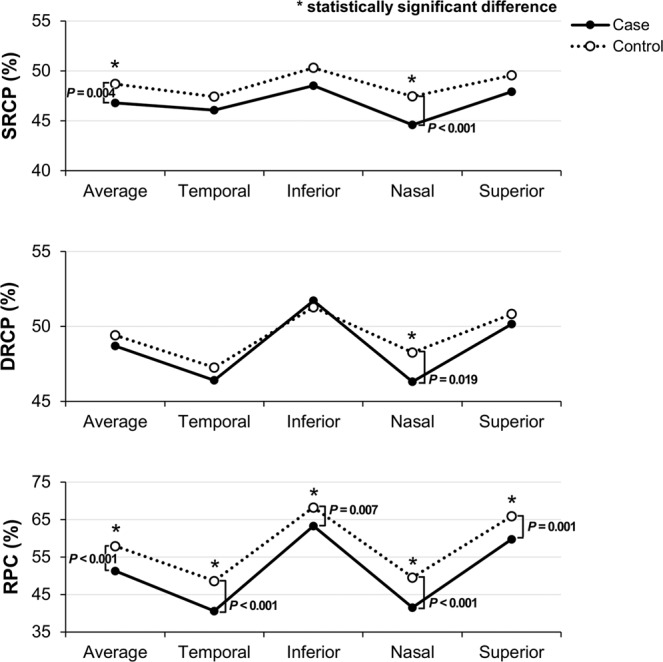
Comparison of the microvascular density between the superficial, deep retinal capillary plexus, and the radial peripapillary capillary segment in healthy controls and case patients with chiasmal compression. Vessel densities of the nasal quadrant of SRCP and DRCP and all quadrants of the RPC segment are significantly reduced in the case group compared to the control group. Asterisks indicate statistically significant differences between the two groups.

When the peripapillary microvascular densities were compared, the average vessel density of the radial peripapillary capillary (RPC) segment in the eyes with chiasmal compression was significantly lower than in the control eyes after adjusting for age and SE in linear regression analysis (51.3% ± 5.7% vs. 57.9% ± 3.2%, *P* < 0.001). Based on a sector-wise comparison of the peripapillary microvascular densities, the vessel density of the RPC segment in the eyes with chiasmal compression was significantly lower than in the control eyes in all four quadrants (Table 3, Fig. 2).

**Table 3:** Comparison of microvascular density between the superficial, deep retinal capillary plexus and RPC segment in the healthy control and patients with chiasmal compression.

	Average	Center	Temporal	Inferior	Nasal	Superior
**SRCP, % area**						
Case	**46.80 ± 2.7**	19.36 ± 4.9	46.08 ± 2.9	48.53 ± 4.8	**44.58 ± 2.5**	47.92 ± 4.2
Control	**48.69 ± 2.6**	18.98 ± 4.4	47.42 ± 2.9	50.31 ± 4.0	**47.45 ± 2.6**	49.56 ± 3.3
*P* Value	0.004*	0.654	0.058	0.101	< 0.001*	0.080
**DRCP, % area**						
Case	48.71 ± 3.8	15.36 ± 4.5	46.40 ± 3.2	51.71 ± 6.4	**46.31 ± 3.6**	50.16 ± 4.6
Control	49.41 ± 2.5	15.78 ± 4.6	47.25 ± 3.0	51.28 ± 5.0	**48.25 ± 2.9**	50.82 ± 3.4
*P* Value	0.454	0.703	0.306	0.682	0.019*	0.633
**RPC, % area**						
Case	**51.31 ± 5.7**	**29.31 ± 10.3**	**40.61 ± 7.6**	**63.29 ± 8.7**	**41.52 ± 8.5**	**59.79 ± 8.8**
Control	**57.94 ± 3.2**	**34.85 ± 7.6**	**48.60 ± 5.3**	**68.25 ± 5.0**	**49.54 ± 5.6**	**65.88 ± 4.8**
*P* Value	<0.001*	0.019*	<0.001*	0.007*	<0.001*	0.001*

Data are given as mean ± standard deviation. SRCP = superficial retinal capillary plexus; DRCP = deep retinal capillary plexus; RPC = radial peripapillary capillary. *Statistically significant. Linear regression values adjusted for age and spherical equivalent. P Values were corrected by Bonferroni’s correction due to multiple testing.

### Correlations of vessel density with pRNFL thickness, GCC thickness, and visual field defects

**Figure 3:**
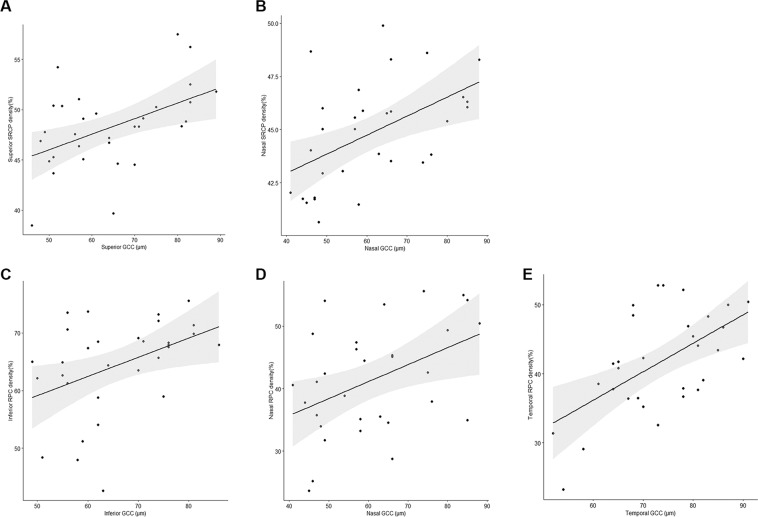
The scatterplot shows the correlation between each quadrant microvascular density and sectoral ganglion cell layer complex (GCC) in patients with chiasmal compression. (**A**) Correlation between the superior superficial retinal capillary plexus (SRCP) and the superior GCC (r = 0.482, *P* = 0.006); (**B**) Correlation between the nasal SRCP and the nasal GCC (r = 0.510, *P* = 0.003); (**C**) Correlation between the inferior radial peripapillary capillary (RPC) and the inferior GCC (r = 0.419, *P* = 0.019); (**D**) Correlation between the nasal RPC and the nasal GCC (r = 0.435, *P* = 0.015); (**E**) Correlation between the temporal RPC and the temporal GCC (r = 0.578, *P* = 0.001). The black line indicates the linear regression line and the dark gray area the corresponding 95% CI.

**Figure 4:**
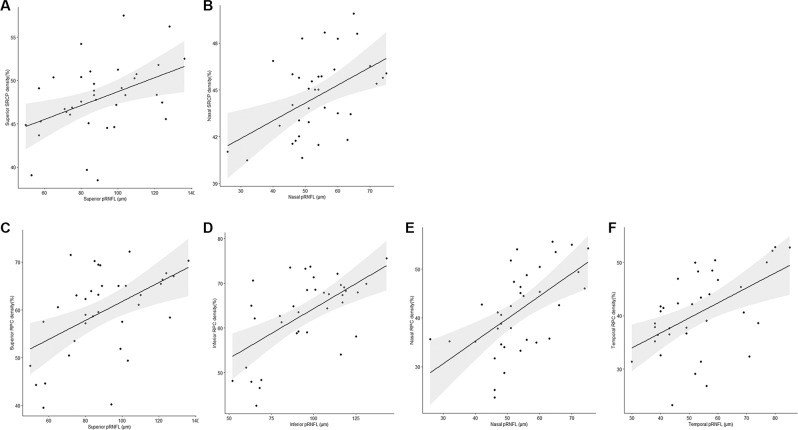
The scatterplot shows the correlation between each quadrant microvascular density and sectoral peripapillary retinal nerve fiber layer (pRNFL) in patients with chiasmal compression. (**A**) Correlation between the superior superficial retinal capillary plexus (SRCP) and the superior pRNFL (r = 0.438, *P* = 0.008); (**B**) Correlation between the nasal SRCP and the nasal pRNFL (r = 0.490, *P* = 0.002); Correlation between all the quadrants of radial peripapillary capillary (RPC) and the corresponding areas of pRNFL, respectively, (**C**, superior, r = 0.507, *P* = 0.002; (**D**) inferior, r = 0.599, *P* < 0.0001; E, nasal, r = 0.584, *P* < 0.0001; F, temporal, r = 0.522, *P* = 0.001). The black line indicates the linear regression line and the dark gray area the corresponding 95% CI.

**Figure 5:**
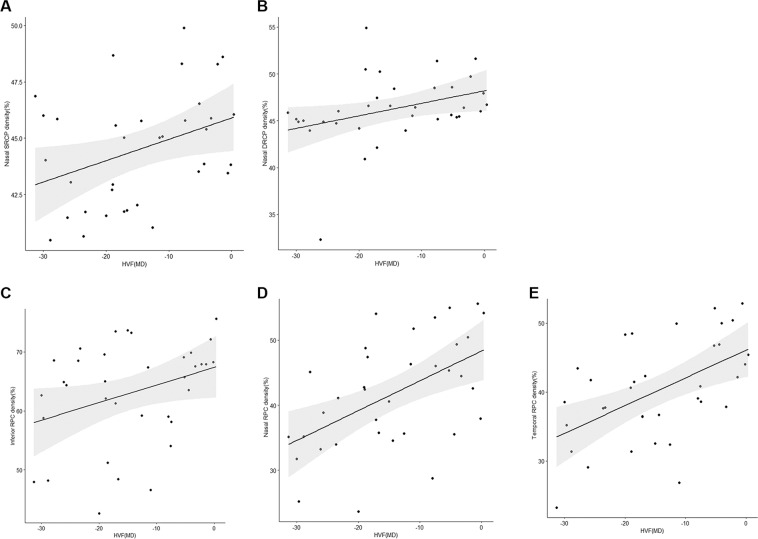
The scatterplot shows the correlation between each quadrant microvascular density and visual field defects in patients with chiasmal compression. (**A**) Correlation between the nasal superficial retinal capillary plexus and the visual field defects (r = 0.369, *P* = 0.029); (**B**) Correlation between the nasal deep retinal capillary plexus and the visual field defects (r = 0.357, *P* = 0.036); (**C**) Correlation between the inferior radial peripapillary capillary (RPC) and the visual field defects (r = 0.337, *P* = 0.048); (**D**) Correlation between the nasal RPC and the visual field defects (r = 0.527, *P* = 0.001); (**E**) Correlation between the temporal RPC and the visual field defects (r = 0.538, *P* = 0.001). The black line indicates the linear regression line and the dark gray area the corresponding 95% CI.

The relationship between vessel densities and other parameters, including the pRNFL thickness, the GCC thickness, and the visual field defects, have been analyzed in patients with chiasmal compression. When the inner retinal layers were analyzed by sectors, pRNFL and GCC thicknesses in all quadrants showed a significant correlation with the vessel densities in the RPC segment. Additionally, the superior and nasal quadrant pRNFL and GCC thicknesses showed significant correlations with the vessel densities of the SRCP. However, we have not detected any significant correlation between the sector vessel densities in the DRCP area and the inner retinal layers (Figs. 3 and 4). A significant relationship between visual field defects and the vessel densities of the nasal SRCP and DRCP, and all quadrants excepting superior quadrant of RPC segment was detected (Table 4, Fig. 5).

**Table 4:** Spearman coefficient correlation of peripapillary and macular vessel density, inner retinal layer thickness, and visual field defects.

	GCC		pRNFL		Visual field^†^, MD	
	*R*	*P* Value	*R*	*P* Value	*R*	*P* Value
Superior sector	Superior GCC thickness		Superior pRNFL thickness			
SRCP vessel density	**0**.482*	**0**.**006**	**0**.438*	**0**.**008**	0.294	0.086
DRCP vessel density	0.156	0.404	0.111	0.521	0.150	0.388
RPC vessel density	0.230	0.214	**0**.507*	**0**.**002**	0.218	0.209
Inferior sector	Inferior GCC thickness		Inferior pRNFL thickness			
SRCP vessel density	0.014	0.939	0.150	0.383	0.160	0.357
DRCP vessel density	0.223	0.229	0.113	0.511	0.018	0.919
RPC vessel density	**0**.419*	**0**.**019**	**0**.599*	**<0**.**001**	**0**.**337**	**0**.048*
Nasal sector	Nasal GCC thickness		Nasal pRNFL thickness			
SRCP vessel density	**0**.510*	**0**.**003**	**0**.490*	**0**.**002**	**0**.**369**	**0**.029*
DRCP vessel density	0.157	0.398	0.294	0.082	**0**.**357**	**0**.036*
RPC vessel density	**0**.435*	**0**.**015**	**0**.584*	**<0**.**001**	**0**.**527**	**0**.001*
Temporal sector	Temporal GCC thickness		Temporal pRNFL thickness			
SRCP vessel density	0.04	0.834	0.143	0.411	0.144	0.416
DRCP vessel density	0.064	0.738	0.034	0.847	0.142	0.421
RPC vessel density	**0**.578*	**0**.**001**	**0**.522*	**0**.**001**	**0**.**538**	**0**.001*

SRCP = superficial retinal capillary plexus; DRCP = deep retinal capillary plexus; RPC = radial peripapillary capillary; GCC = ganglion cell layer complex; pRNFL = peripapillary retinal nerve fiber layer; MD = mean deviation. * Statistically significant. ^†^Humphrey Field Analyzer using the 30-2 SITA-standard protocol.

## Discussion

In our study, the nasal quadrant vessel densities of the SRCP and DRCP in patients with chiasmal compression were decreased compared with the healthy control group after adjusting for age and SE. In the RPC segment, vessel densities in all peripapillary quadrants were decreased compared with the healthy control group. Few studies have examined eyes with chiasmal compression using OCT-A^13^. Higashiyama *et al*. have reported that only peripapillary areas with a decrease in retinal perfusion correlated with the location of the visual field defects due to chiasmal compression, but not the macular areas^13^. We have found that all quadrants in the peripapillary area and the nasal sector of the macula had significantly lower microvascular densities in the eyes with chiasmal compression than in control eyes. It is widely known that bitemporal hemianopsia due to chiasmal compressive lesions is related with a loss of retinal ganglion cell axons in the nasal hemiretina which generally enter the optic disc via temporal and nasal sides^14^. Damage to the ganglion cells due to compressive chiasmal lesions may decrease the metabolic activity and affect the nutrient demand and supply, leading to changes in the retinal vascular structures, especially in the corresponding area.

Previous studies using OCT have also reported pRNFL loss and macular changes, such as loss of ganglion cells in chiasmal compression^6,15,16^. Our study has demonstrated a significant thinning of the pRNFL and GCC in the eyes with chiasmal compression compared with the controls, consistent with the foregoing studies.

We found significant correlations between the peripapillary and macular vessel densities and the degree of visual field defects in the eyes with chiasmal compression. In sector analysis, only the nasal quadrant of the SRCP and the DRCP and the inferior, nasal, and temporal quadrants of the RPC segment have been significantly correlated with visual field defects. As mentioned above, Higashiyama *et al*. have reported that the peripapillary retinal perfusion was decreased in the corresponding area of the visual field defects in chiasmal compression^13^. Significant correlations between the vessel densities in the RPC segment and BCVA, visual field defects, and pRNFL thickness have also been reported in eyes diagnosed with ischemic optic neuropathy^10^, similar to our results. In other optic neuropathies associated with neuromyelitis optica, Kwapong *et al*. have also reported that decreased microvascular densities in the macular area were correlated with visual acuity^11^.

We have found significant correlations between vessel densities of RPC segment in all quadrants and OCT parameters including pRNFL and GCC thicknesses in the corresponding quadrants, similar to a previous study investigating other types of optic neuropathy as mentioned above^10^. We have also found significant correlations between the superior and nasal quadrant vessel densities of the SRCP and the corresponding regions of pRNFL and GCC thicknesses. This result indicates that the microvascular alteration in the nasal and superior sectors of the superficial plexus reflected a loss of retinal nerve fibers and ganglion cells in the corresponding areas of chiasmal compression. The first affected area may have led to irreversible changes in retinal structures.

New technologies for the measurement of vessel density have been reported since the development of OCT-A. Previously, OCT-A images were converted into binary images using Image J. The total number of pixels occupied by vessels was then divided by the total number of pixels in the entire image and the value was expressed as a ratio^17,18^. However, automatic quantification using other OCT-A instrument software has demonstrated excellent repeatability and reproducibility^19,20^. Data related to age-related vessel density mapping using OCT-A prototype software (AngioAnalytics, automatic quantification tool) in healthy subjects have been regarded as normative data within the range of 56 to 61 percent^20^. In the present study, all parafoveal areas of vascular density were 47 to 51% in the control group using the internal computer software (IMAGEnet 6) based on swept-source OCT-A, with a mean age of 50 years. However, other studies have reported that the vessel density calculated as pixels using binarized images with Image J external software from the same swept-source OCT-A instrument was 38 to 45 percent^18^. The discrepancy has been attributed to differences in software and analytical methods.

Our study presents certain limitations. First, our study has been a cross-sectional study. We could not comment on the prognostic value of vessel densities in disease progression. Longitudinal studies are needed to investigate the relationship between vessel density and changes in other functional and anatomical parameters in chiasmal copressionafter decompression surgery. Second, age, refractive error, and axial length may be important confounding factors in intraretinal layer analysis using OCT^21^. Therefore, adjustment for these factors is necessary for the analysis. In this study, we did not routinely check axial length. Instead, SE refractive error along with age was adjusted in the analysis. Third, the small sample size in each group of this study prevented further subgroup analysis of patients with compressive optic neuropathy diagnosed with different disease severities. Fourth, our data were derived from a single center and involved a population of Asian ethnicity. Consequently, some of our results may not be appropriate for other races. Fifth, we did not perform fluorescein angiography in patients with chiasmal compression. Fluorescein angiography is the gold standard and may also highlight a very slow flow imaging with OCT-A. Further prospective and comprehensive studies are required to elucidate the pathophysiology of microvascular changes in compressive optic neuropathy and the effect of decompression on this changes. The prognostic value of OCT-A in chiasmal compressions also needs to be determined in further studies.

However, this study does provide the first quantitative description of microvascular changes in patients with optic chiasmal compression compared with healthy controls using the swept-source OCT-A automated software. Vessel densities measured by OCT-A might facilitate our understanding of the diverse pathologic changes in optic atrophy caused by optic chiasmal compression.

In conclusion, we have demonstrated significant microvascular alterations around the peripapillary area and the nasal sector of the macula in patients with chiasmal compression. Microvascular alterations in the RPC segment of eyes with chiasmal compression have been strongly correlated with the pRNFL, the GCC, and visual field defects. In the macular area, microvascular densities in the superior and nasal quadrants were significantly correlated with the corresponding area of pRNFL and GCC thicknesses. This study confirmed that chiasmal compression caused by brain tumors not only induced the loss of ganglion cells but also triggered intra-retinal microvascular changes.

## Patients and Methods

This cross-sectional study has been approved by the Institutional Review Board (2018-04-131) of Samsung Medical Center (Seoul, Republic of Korea). Written informed consent has been obtained from all participants and normal controls before any study procedures were initiated, and data collection followed the tenets of the Declaration of Helsinki. This study included patients with chiasmal compression caused by brain tumors and healthy controls who visited the Department of Neuro-ophthalmology and Neurosurgery of Samsung Medical Center from January 1, 2017 to July 31, 2018. The study involved patients with evidence of optic neuropathy or chiasmal syndrome based on compatible visual field defects or decreased visual acuity and chiasmal compressive tumors confirmed by magnetic resonance imaging. Only a single eye (the one with the worse visual field defects based on mean deviation values) of each patient at 4–12 months after tumor resection has been selected for analysis. Patients and healthy controls with ophthalmic disease (glaucoma, a refractive error greater than 6.0 diopters of spherical equivalent as high myopia and hyperopia or 3.0 diopters of astigmatism in either eye, amblyopia, epiretinal membrane, age-related macular degeneration, diabetic retinopathy, retinal artery/vein occlusion, optic neuritis, and other ischemic optic neuropathy), previous retinal surgery which may affect vessel density, and those with known systemic or inflammatory diseases (cancer, multiple sclerosis, etc.) have been excluded. Healthy controls were required to have normal optic discs, normal thickness of the intra-retinal layers, and intraocular pressure (IOP) < 21 mm. Fundus photography and Cirrus HD-OCT (Carl Zeiss Meditec AG, Jena, Germany) were performed for all eyes. The visual field perimetry for patients with chiasmal compression has been performed with a Humphrey Field Analyzer using the 30-2 SITA-standard protocol (Humphrey 740 Visual Field Analyzer, Carl Zeiss Meditec Inc. Dublin, CA, USA). Only reliable visual fields ( ≤ 33% false positives or false negatives, fixation losses < 20%) have been considered. The mean deviation was used for the analysis.

### Optical coherence tomography

All patients and healthy controls underwent Cirrus HD-OCT. The pRNFL thickness has been determined using the optic disc cube 200 × 200 protocol with Cirrus software. This protocol generates a cube of data through a 6-mm-square grid. A 3.46-mm-diameter circle is automatically centered on the optic disc. This analytical protocol yields an average RNFL thickness, maps four quadrants (temporal, inferior, nasal, and superior), and classifies results compared with an internal normative database. Only scans with a signal strength ≥ 6 without motion artifacts have been included. For the GCC analysis, the thickness of the ganglion cell layer and the inner plexiform layers (IPL) were evaluated. The GCC thickness was automatically measured at various locations around the fovea (temporal, inferior, nasal, and superior) and the thickness of each of the four quadrants, was calculated.

### Optical coherence tomography angiography and image analysis

**Figure 6:**
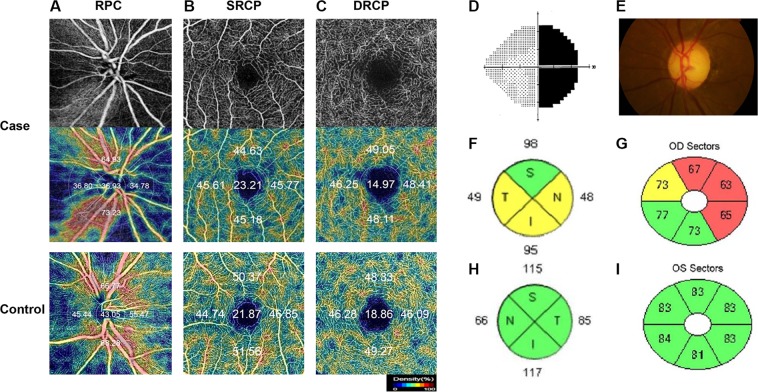
Representative case images of patients with chiasmal compression and control images of a healthy eye. OCT-A en-face images of patients with pituitary adenoma who underwent transsphenoidal tumor resection (Top) and color-coded density maps of the patients (Middle) and a healthy control (Bottom) in the radial peripapillary capillary (RPC) segment (**A**), the superficial retinal capillary plexus (SRCP) (**B**), and the deep retinal capillary plexus (DRCP). (**C**) Color-coded density maps show automated measurements of the vessel density with percentages via auto-segmentation. Vessel densities of the RPC segment and SRCP were lower in the patient cases than in the control. Figures (**D**–**G**) show the preoperative visual field with temporal hemianopsia (**D**), disc photography with band atrophy (**E**), the peripapillary retinal nerve fiber layer thickness map (**F**), and the ganglion cell layer complex thickness map (**G**) in patients with chiasmal compression. Both maps (**F**,**G**) show significant thinning. Figures H and I show the peripapillary retinal nerve fiber layer thickness map (**H**) and the ganglion cell layer complex thickness map (**I**) in the healthy control.

The retinal and peripapillary microvasculature have been analyzed using a Topcon OCT instrument (DRI OCT Triton Plus) for all patients and healthy controls. All OCT-A imaging was performed 4–12 months after tumor resections. The Triton swept-source OCT uses a wavelength of 1,050 nm with a scan speed of 100,000 A-scans per second. For each field scan, three repeated B-scans obtained from 500 uniformly spaced locations have been sequentially acquired in order to verify the repeatability of vessel density measurement. Each B-scan consisted of 500 A-scans and the interscan time between repeated B-scans was about 5 ms, accounting for the mirror scan duty cycle. The instrument uses an active eye tracker which follows fixation movement. It detects blinking and adjusts the scan position accordingly, thereby reducing motion artifacts during OCT-A image generation. Each patient underwent two imaging sessions consisting of a 4.5 × 4.5 mm diameter peripapillary scan centered on the optic disc and a 3.0 × 3.0 mm diameter perifoveal scan centered on the macula. The SRCP and DRCP were separated automatically via layer segmentation with the OCT instrument software (IMAGEnet 6 V.1.14.8538). It resulted in automated segmentation and offers several preset reference boundaries for en-face projection, with a final rendered depth scale of 2.6 µm/voxel. Swept source OCT-A evaluation included vessel density, foveal avascular zone area, presence of vascular abnormalities such as dilated endings of the capillaries and a number of CC flow voids. The SRCP extended from 3 µm below the internal limiting membrane (ILM) to 15 µm below the IPL, while the DRCP extended from 15 to 70 µm below the IPL, according to a previously validated method by Park *et al*.^22^. The RPC segment extended from the ILM to the posterior boundary of the RNFL. Vessel density has been defined as the percentage of the area occupied by vessels in a localized region. The software automatically fitted an Early Treatment Diabetic Retinopathy Study (ETDRS) circular to the center of optic disc and foveal avascular zone, generated vessel density for each layer with high repeatability and reproducibility^19,23^. The foveal margin was used to calculate the average vessel density. Five areas (center, temporal, inferior, nasal, and superior) dividing the center of the macula and disc are displayed (Fig. 6). The blood vessel density of each area has been indicated as a percentage. All participants underwent both OCT-A and Cirrus HD-OCT imaging on the same day. We have excluded eyes with low image quality < 40 and those showing a partial decrease in image intensity. Any large eye movements during image capture were reflected in motion artifacts of more than three lines. The eyes were also excluded if any discontinuities of the blood vessels in the OCT angiography image were seen.

### Statistical analysis

The data are presented as the mean ± standard deviation (SD). The BCVA was converted to a logMAR scale. The Student’s t-test and the Wilcoxon rank sum test were used to compare the differences in the demographic data between patients with chiasmal compression and controls. A Bonferroni correction was used for post-hoc analysis. Linear regression analysis was conducted after adjusting for age and SE to compare vessel densities between the patient and healthy control groups. Spearman’s correlation coefficients were calculated to analyze the correlations between visual field defects, intra-retinal layer thicknesses, and microvascular densities. A *P* value less than 0.05 was considered statistically significant. All statistical analyses were performed with STATA version 14.1 (Stata Corp LP, College Station, TX, USA).

## Data Availability

The datasets generated during and/or analyzed during the current study are available from the corresponding author upon reasonable request.

## References

[1] Danesh-Meyer HV (2006). Relationship between retinal nerve fiber layer and visual field sensitivity as measured by optical coherence tomography in chiasmal compression. Invest Ophthalmol Vis Sci.

[2] Garcia T (2014). Prognostic value of retinal nerve fiber layer thickness for postoperative peripheral visual field recovery in optic chiasm compression. Journal of neurosurgery.

[3] Phal PM (2016). Assessment of Optic Pathway Structure and Function in Patients With Compression of the Optic Chiasm: A Correlation With Optical Coherence Tomography. Invest Ophthalmol Vis Sci.

[4] Tieger MG (2017). Ganglion Cell Complex Loss in Chiasmal Compression by Brain Tumors. Journal of neuro-ophthalmology: the official journal of the North American Neuro-Ophthalmology Society.

[5] Monteiro ML, Cunha LP, Costa-Cunha LV, Maia OO, Oyamada MK (2009). Relationship between optical coherence tomography, pattern electroretinogram and automated perimetry in eyes with temporal hemianopia from chiasmal compression. Invest Ophthalmol Vis Sci.

[6] Moon CH, Hwang SC, Ohn YH, Park TK (2011). The time course of visual field recovery and changes of retinal ganglion cells after optic chiasmal decompression. Invest Ophthalmol Vis Sci.

[7] Ohkubo S (2012). Relationship between macular ganglion cell complex parameters and visual field parameters after tumor resection in chiasmal compression. Japanese journal of ophthalmology.

[8] Yarmohammadi A (2016). Relationship between Optical Coherence Tomography Angiography Vessel Density and Severity of Visual Field Loss in Glaucoma. Ophthalmology.

[9] Liu CH, Kao LY, Sun MH, Wu WC, Chen HS (2017). Retinal Vessel Density in Optical Coherence Tomography Angiography in Optic Atrophy after Nonarteritic Anterior Ischemic Optic Neuropathy. Journal of ophthalmology.

[10] Augstburger E, Zeboulon P, Keilani C, Baudouin C, Labbe A (2018). Retinal and Choroidal Microvasculature in Nonarteritic Anterior Ischemic Optic Neuropathy: An Optical Coherence Tomography Angiography Study. Investigative ophthalmology & visual science.

[11] Kwapong WR (2018). Altered Macular Microvasculature in Neuromyelitis Optica Spectrum Disorders. American journal of ophthalmology.

[12] Mammo Z (2016). Quantitative Optical Coherence Tomography Angiography of Radial Peripapillary Capillaries in Glaucoma, Glaucoma Suspect, and Normal Eyes. American journal of ophthalmology.

[13] Higashiyama T, Ichiyama Y, Muraki S, Nishida Y, Ohji M (2016). Optical Coherence Tomography Angiography of Retinal Perfusion in Chiasmal Compression. *Ophthalmic Surg Lasers Imaging*. Retina.

[14] Kanamori A (2004). Optical coherence tomography detects characteristic retinal nerve fiber layer thickness corresponding to band atrophy of the optic discs. Ophthalmology.

[15] Vuong LN, Hedges TR (2017). Ganglion cell layer complex measurements in compressive optic neuropathy. Curr Opin Ophthalmol.

[16] Sun M, Zhang Z, Ma C, Chen S, Chen X (2017). Quantitative analysis of retinal layers on three-dimensional spectral-domain optical coherence tomography for pituitary adenoma. PloS one.

[17] Ghasemi Falavarjani K (2016). Swept-Source Optical Coherence Tomography Angiography Of The Optic Disk In Optic Neuropathy. Retina.

[18] Al-Sheikh M, Ghasemi Falavarjani K, Akil H, Sadda SR (2017). Impact of image quality on OCT angiography based quantitative measurements. International journal of retina and vitreous.

[19] Al-Sheikh M, Tepelus TC, Nazikyan T, Sadda SR (2017). Repeatability of automated vessel density measurements using optical coherence tomography angiography. The British journal of ophthalmology.

[20] Coscas F (2016). Normative Data for Vascular Density in Superficial and Deep Capillary Plexuses of Healthy Adults Assessed by Optical Coherence Tomography Angiography. Invest Ophthalmol Vis Sci.

[21] Park KA, Oh SY (2015). Retinal nerve fiber layer thickness in prematurity is correlated with stage of retinopathy of prematurity. Eye (London, England).

[22] Park JJ, Soetikno BT, Fawzi AA (2016). Characterization of The Middle Capillary Plexus Using Optical Coherence Tomography Angiography In Healthy and Diabetic Eyes. Retina (Philadelphia, Pa.).

[23] Forte, R., Haulani, H., Dyrda, A. & Jurgens, I. Swept source optical coherence tomography angiography in patients treated with hydroxychloroquine: correlation with morphological and functional tests. *The British journal of ophthalmology* (2019). 10.1136/bjophthalmol-2018-313679 30842084

